# Study protocol: a cluster randomized controlled trial of web-based decision support tools for increasing *BRCA1/2* genetic counseling referral in primary care

**DOI:** 10.1186/s12913-018-3442-x

**Published:** 2018-08-13

**Authors:** Thomas B. Silverman, Alejandro Vanegas, Awilda Marte, Jennie Mata, Margaret Sin, Juan Carlos Rodriguez Ramirez, Wei-Yann Tsai, Katherine D. Crew, Rita Kukafka

**Affiliations:** 1 Department of Biomedical Informatics, Columbia University, New York, NY USA; 2 Department of Medicine, Columbia University, New York, NY USA; 3 Department of Biostatistics, Columbia University, New York, NY USA; 4 Herbert Irving Comprehensive Cancer Center, Columbia University Medical Center, New York, NY USA

**Keywords:** Breast Cancer prevention, Decision support, Risk communication, Genetic testing

## Abstract

**Background:**

*BRCA1* and *BRCA2* mutations confer a substantial breast risk of developing breast cancer to those who carry them. For this reason, the United States Preventative Services Task Force (USPSTF) has recommended that all women be screened in the primary care setting for a family history indicative of a mutation, and women with strong family histories of breast or ovarian cancer be referred to genetic counseling. However, few high-risk women are being routinely screened and fewer are referred to genetic counseling. To address this need we have developed two decision support tools that are integrated into clinical care.

**Method:**

This study is a cluster randomized controlled trial of high-risk patients and their health care providers. Patient-provider dyads will be randomized to receive either standard education that is supplemented with the patient-facing decision aid, *RealRisks*, and the provider-facing *Breast Cancer Risk Navigation Toolbox (BNAV)* or standard education alone. We will assess these tools’ effectiveness in promoting genetic counseling uptake and informed and shared decision making about genetic testing.

**Discussion:**

If found to be effective, these tools can help integrate genomic risk assessment into primary care and, ultimately, help expand access to risk-appropriate breast cancer prevention options to a broader population of high-risk women.

**Trial registration:**

This trial is retrospectively registered with ClinicalTrials.gov Identifier: NCT03470402: 20 March 2018.

## Background

Breast and ovarian cancers confer significant morbidity and mortality to women in the United States, and their development is strongly influenced by genetic predisposition [1, 2]. Hereditary breast and ovarian cancer syndrome (HBOC) is an inherited condition that is most often associated with mutations in the *BRCA1* and *BRCA2* (*BRCA1/*2) genes [3]. Carriers of these pathogenic mutations face a lifetime breast cancer risk of 60–80% and a lifetime ovarian cancer risk of 20–40% [4]. Inherited *BRCA1/2* mutations account for an estimated 2–7% of breast cancers and 10–15% of ovarian cancers [3, 5, 6]. Approximately one out of every three to four hundred individuals in the general population carry a *BRCA1/2* mutation [7].

While mutation carriers are at a substantially higher risk for developing breast and ovarian cancers than the general population, preventative actions can reduce a carrier’s cancer risk by up to 90% once she is identified [8]. Such risk-reducing strategies include intensive breast cancer screening with mammography and breast MRI, [9–11] risk-reducing surgeries (prophylactic mastectomy, bilateral salpingo-oophorectomy [BSO]), [12–18] and chemoprevention [19, 20]. Risk-reducing BSO among women with known *BRCA1*/*2* mutations is further associated with a reduction in cancer-specific and all-cause mortality [15, 18].

In order to promote the identification of women carrying *BRCA1/2* mutations, the United States Preventive Services Task Force (USPSTF) recommends that primary care providers screen asymptomatic women for an increased *BRCA1/2* mutation risk [21]. Women who screen positive should receive genetic counseling by a trained health care provider and be offered *BRCA1/2* testing if further indicated and desired after counseling [21].

The identification of women whose family history indicates an increased risk for carrying a mutation is based upon a set of “red flags” in the woman’s medical history and that of her family. These red flags include early onset of breast or ovarian cancer, multiple cases of breast or ovarian cancer in the family, bilateral breast cancer, male breast cancer, Ashkenazi Jewish decent (1 in 40 prevalence of *BRCA1/2* founder mutations), or a previously identified *BRCA1/2* mutation in the family [7].

Despite the USPSTF recommendation and the increasing availability of genetic testing for hereditary cancer syndromes, many women who are at an increased risk of carrying *BRCA1/2* mutations are never identified and are thus unable to receive downstream preventive services [22–25]. In 2016, our research team screened 3055 women who underwent mammographic screening at a large, urban institution [26]. Based on their family histories of breast and ovarian cancer, 12% (*n* = 369) of these women met USPSTF guidelines for *BRCA1/2* genetic testing. Nevertheless, only 4.6% (*n* = 17) of those eligible women had received testing or counseling. Bellcross et al. have reported similar under-utilization of genetic testing services in another health system [22]. In a large sample of women unaffected with breast cancer seen in the primary care setting, 5% met USPSTF guidelines for genetic counseling. Although 91% of these high-risk women reported that they had talked to their health care provider about their family history, only 14% were referred for genetic counseling, and only 4% received *BRCA1/2* genetic testing.

While the prevalence of *BRCA1/2* mutations is similar across most racial and ethnic groups (except Ashkenazi Jews), women from racial/ethnic minority groups and lower education and income levels are less likely to be referred for genetic testing [22, 27, 28]. This lack of risk assessment in minority populations can contribute to further health dipartites and poorer clinical outcomes [29].

Providers find it difficult to assess breast cancer risk and communicate probabilistic risk information to their patients during the primary care encounter [30]. Barriers to family history screening and genetic counseling referral include insufficient knowledge of HBOC and inability to intuit risk, [31–35] lack of time and competing priorities in the primary care encounter, [30, 36] and inadequate reporting of family history in medical records [26, 37]. Patient barriers to discussing and understanding risk include lack of knowledge, low health literacy or numeracy, language barriers, and time constraints [30, 38, 39].

Further research is needed to determine how breast cancer knowledge, the concept of risk—including both general and personalized risk information—and the pros and cons associated with genetic testing are best communicated to women and their providers in order to inform genetic testing decisions and promote risk-appropriate prevention strategies. Improving a patient’s knowledge of breast cancer and genetic testing and the accuracy of her risk perceptions may help her make a more informed choice about pursuing HBOC genetic counseling and testing. Integrating specific and actionable risk information into the clinical encounter may help enable providers to conduct appropriate risk assessments and refer high-risk patients to further testing and downstream services.

## Methods and design

### Aims

The study goal is to expand genetic testing for HBOC to a broader population of high-risk women by prompting appropriate referrals from the primary care setting with the use of an electronic health record-embedded breast cancer risk navigation (*BNAV*) tool [40, 41]. To address patient-related barriers to genetic testing, we developed a web-based decision aid, *RealRisks*, [42, 43] which is designed to improve genetic testing knowledge, accuracy of breast cancer risk perceptions, and self-efficacy to engage in a collaborative dialogue about genetic testing. Specifically, our aim is to conduct a cluster randomized controlled trial to evaluate the effect of patient education with *RealRisks* and *BNAV* compared to patient education alone on promoting appropriate uptake of *BRCA1/2* genetic counseling. Secondarily, we will assess the efficacy if the RealRisks/BNAV intervention with measures of decision process and quality as endpoints.

### Population

This study is being conducted in the outpatient clinics of Columbia University/New York Presbyterian Hospital in New York, NY. These clinics serve about 42,000 adults each year [44]. About 70% of the patients seen at these clinics identify as Hispanic, many from the Dominican Republic and other parts of the West Indies/Caribbean Basin [44]. The majority of patients are covered by Medicaid [44]. We have found that among patients who meet family history criteria for *BRCA1/2* genetic testing, less than 5% have undergone HBOC genetic testing due to the lack of systemic family history screening [26].

The study clinics provide a range of services, including internal medicine, family medicine, gynecology, and family planning. We will engage providers and patients from each of these specialties.

To be eligible, a provider must be a physician, nurse practitioner, physician assistant, or nurse midwife who sees patients in the study clinics and is willing and able to provide informed consent. Patients must see a provider enrolled in the study, be 21 to 75 years of age, have no personal history of breast or ovarian cancer, have never received genetic counseling or testing for HBOC, meet family history criteria for *BRCA1/2* genetic testing based upon a validated family history screener, [27] and be able to provide informed consent in English or Spanish.

### Cluster randomization

Because this study focuses on patient-provider dyads, we will cluster randomize the patients at the provider letter. We will first recruit health care providers, and, upon consenting, these providers will be given a baseline survey and then randomized into either the intervention or control group.

These providers’ patients will then be screened to determine eligibility for the study and for *BRCA1/2* testing. Those who meet eligibility criteria will be recruited and assigned to the same randomization group as their provider. In this way, the patients grouped by provider will form the clusters in this randomized controlled trial.

### Study intervention

The study intervention includes the patient-facing *RealRisks* decision aid coupled with the provider-facing *BNAV* toolbox. These user-centered tools are integrated into clinical workflow, were designed with consistent feedback from members of our target populations, and have been demonstrated to be useable and appropriate [42, 45].

*RealRisks* incorporates constructs from shared decision making and self-determination theory to model patient-provider dialog, communicate numeric concepts central to risk, and engage women in deciding upon a preference-sensitive course of action regarding genetic testing [46, 47]. *RealRisks* was designed to incorporate a patient’s preferences and values in order to improve accuracy of risk perceptions and promote self-efficacy in a way that will support autonomous decision making. Specifically, *RealRisks* was designed to improve genetic testing knowledge, accuracy of breast cancer risk perceptions, and self-efficacy to engage in a collaborative dialogue about breast cancer risk and genetic testing.

**Fig. 1:**
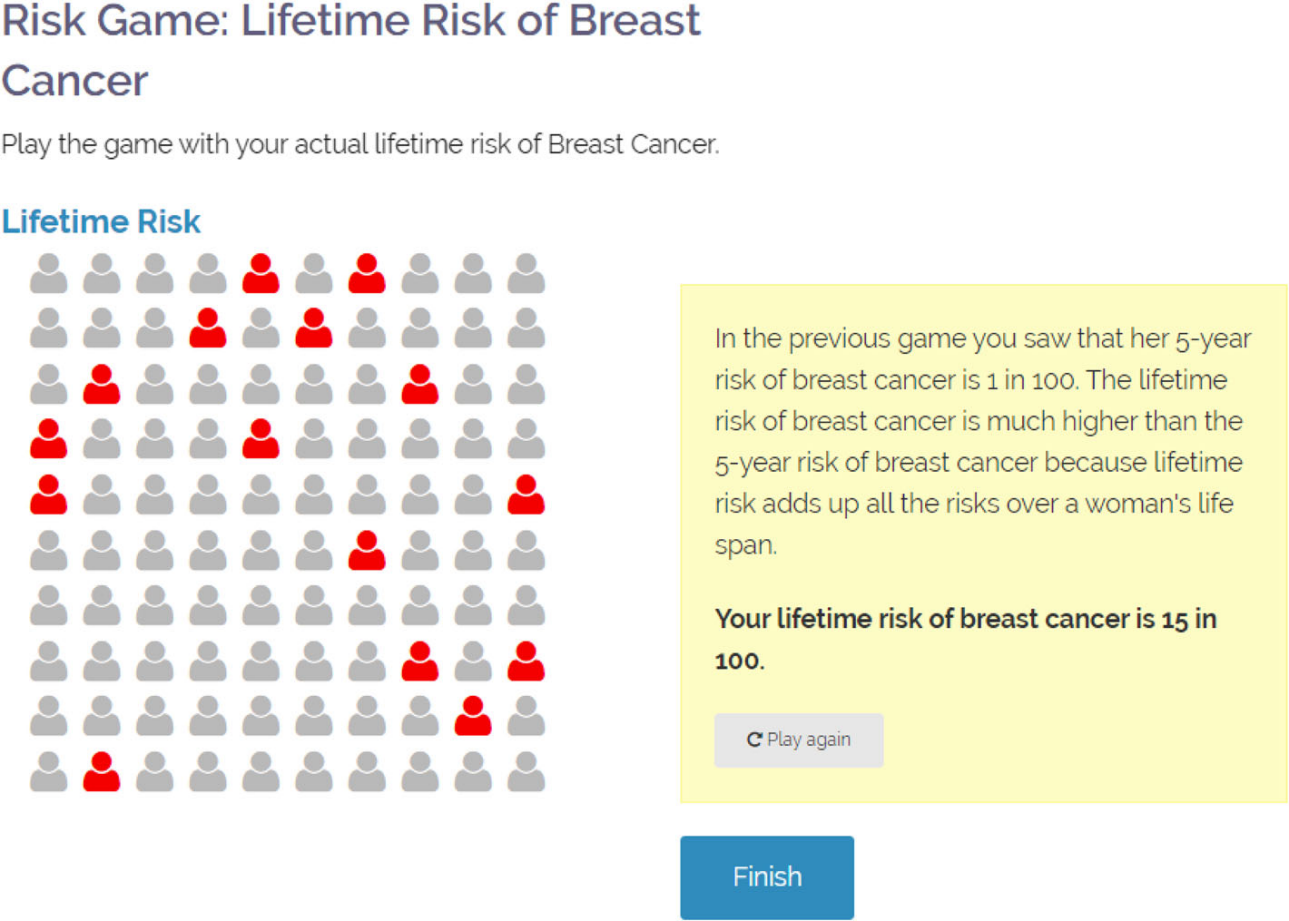
Example of an experience-based, pictographic risk game in *RealRisks*

To accomplish this, *RealRisks* first provides general education using a narrative in which a fictitious character named Rose discusses breast cancer risk, family history, and cancer prevention options with friends, family, and health care providers. Patients can choose to read this narrative in English or Spanish and in graphic novel format (narrative light) or slide presentations (narrative dense). The patient reinforces this information by playing experience-based, pictographic risk games that are embedded into the education modules (Fig. 1).

Next, the patient is instructed to enter her family history data into a family tree. *RealRisks* uses this data to run the BRCAPRO model, which calculates the patient’s personalized five-year breast cancer risk, lifetime breast cancer risk, and probability of carrying a *BRCA1/2* mutation [48, 49]. This information is then presented to the patient through another set of experience-based, pictographic risk games.

Finally, *RealRisks* elicits the patient’s preferences for pursuing *BRCA1/2* genetic testing. This preference elicitation includes the patient’s intention to undergo genetic testing as well as the factors that were important to her in making this decision. Examples of such factors include the increased ability to prevent getting cancer, ease of mind if the test result is negative, costs associated with testing, and privacy or discrimination concerns.

**Fig. 2:**
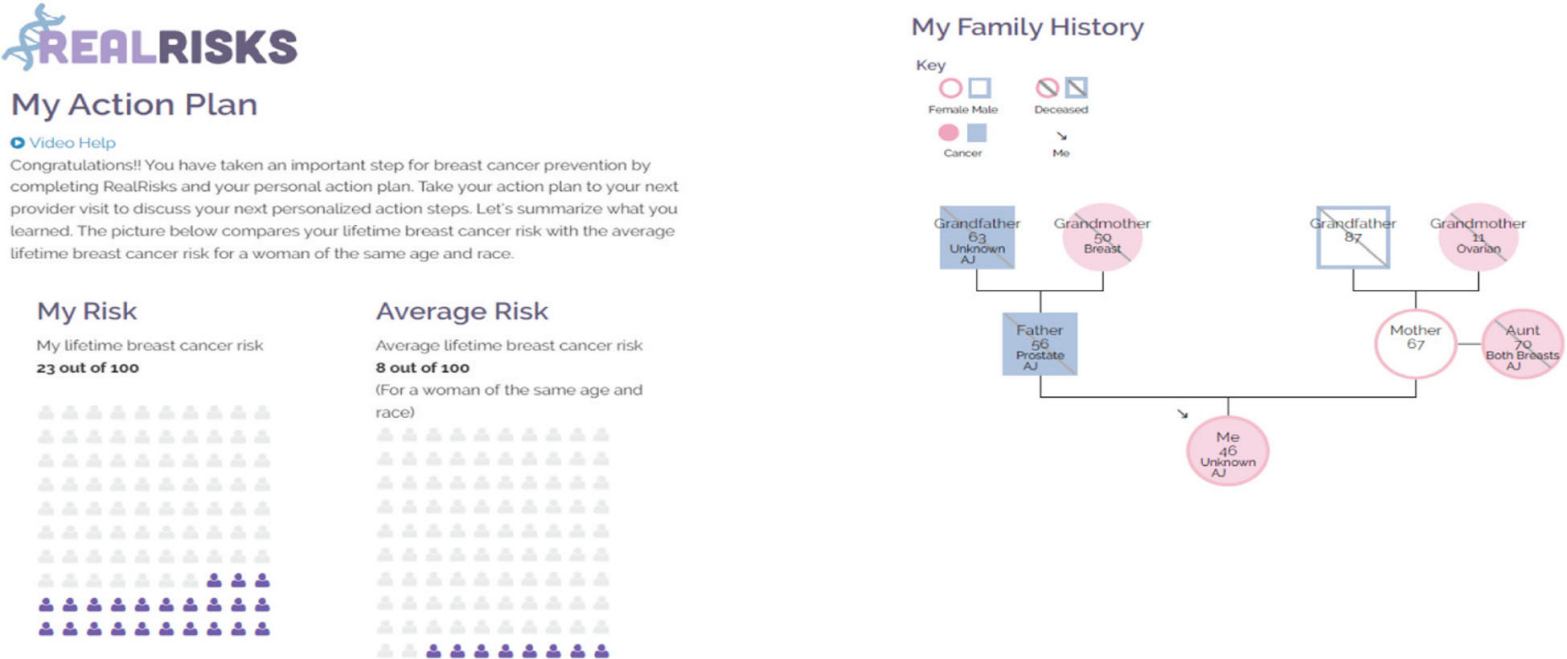
Example summaries provided by the Patient Action Plan

This information is then all summarized for the patient in the Patient Action Plan (Fig. 2), and she is encouraged to bring this action plan to her next appointment with her health care provider.

**Fig. 3:**
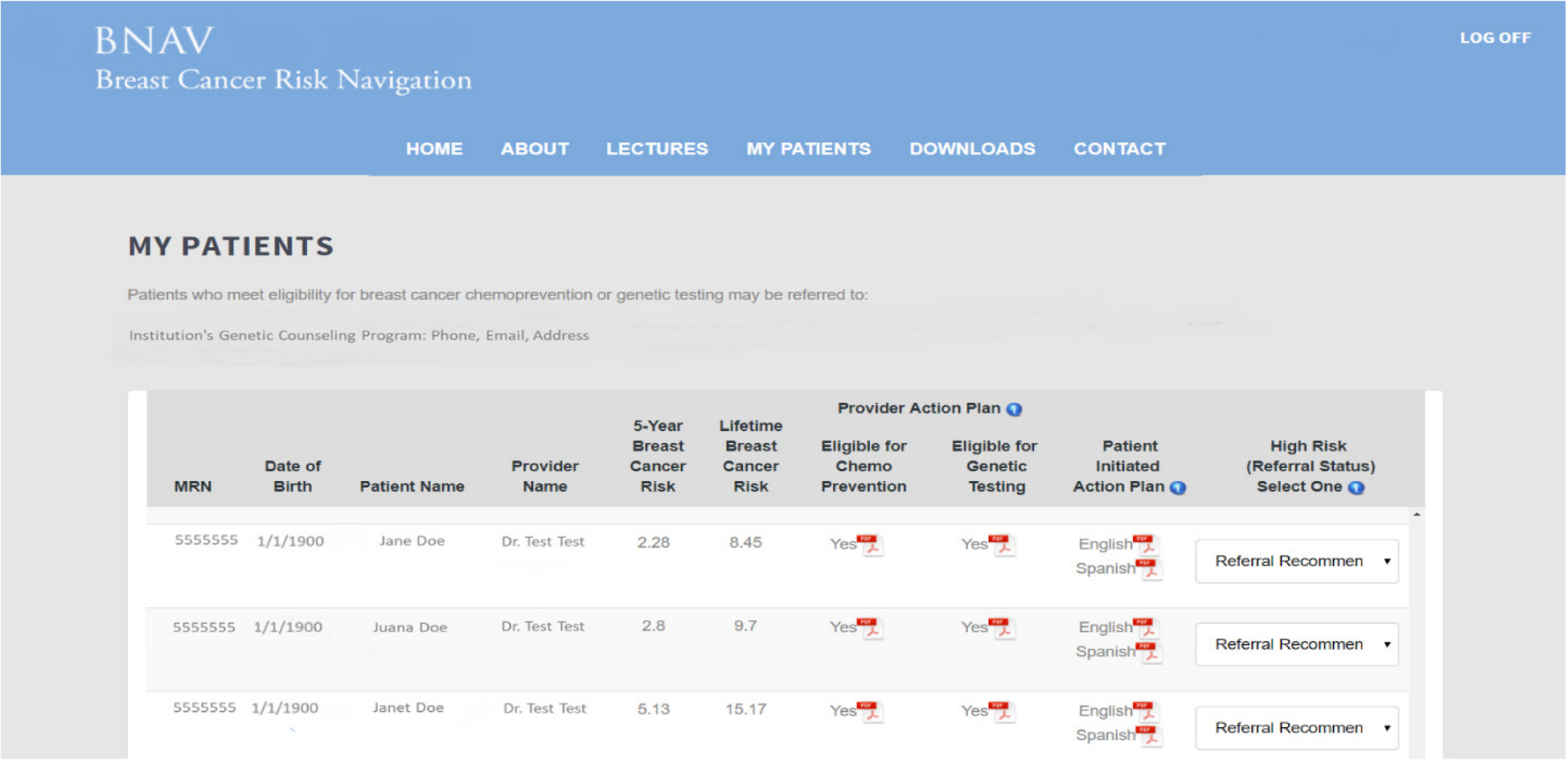
Example of the personalized patient table included in the *BNAV* Toolbox

**Fig. 4:**
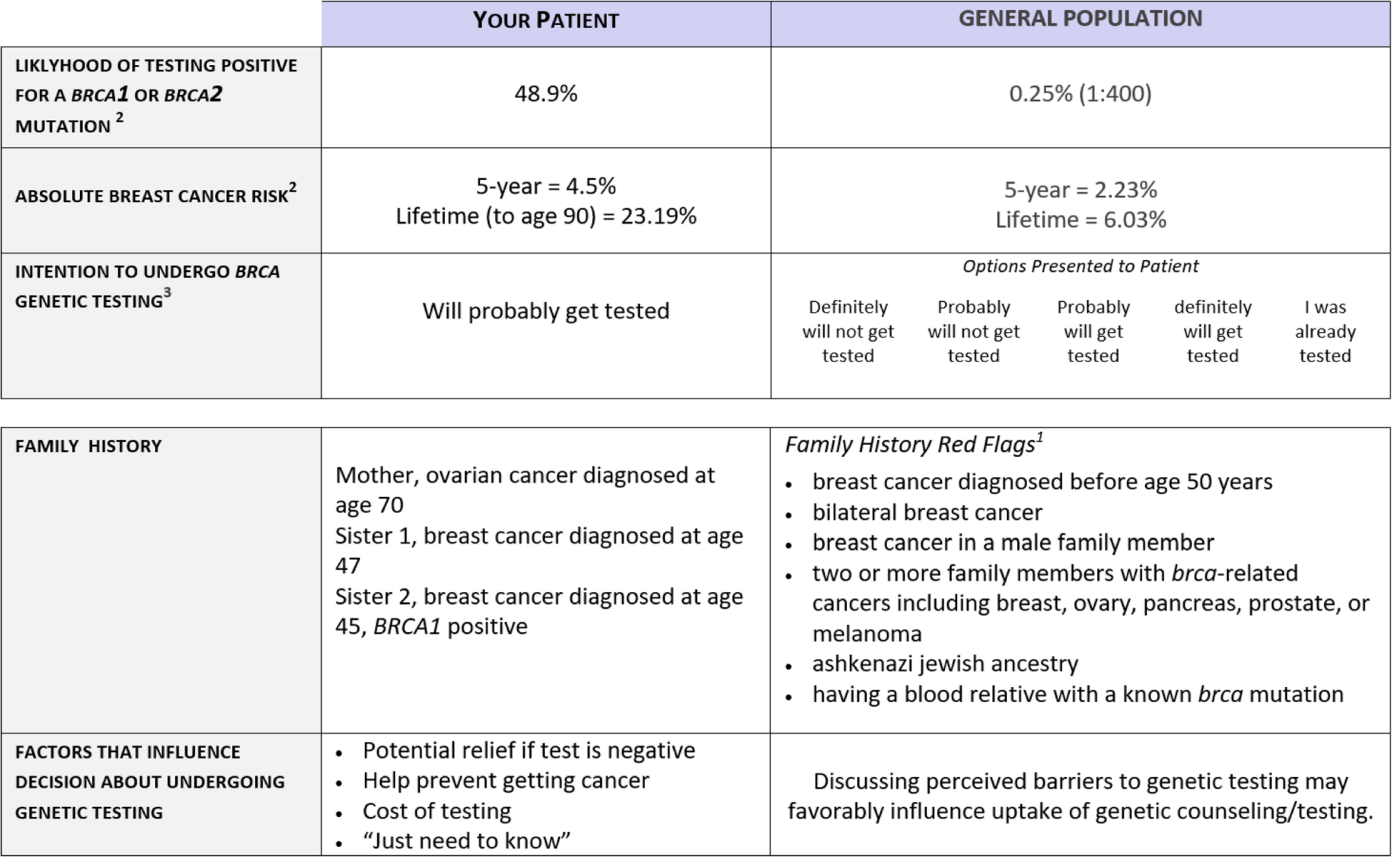
Example of the Provider Action Plan

Once *RealRisks* calculates risks scores based on the patient’s family history data, this information is synchronized into the *BNAV* toolbox for providers. This risk information is displayed in a table that includes the patient’s personalized risk and preference information for each of the provider’s participating patients. In addition, *BNAV* uses this risk data to create a Provider Action Plan, which is similar to the Patient Action Plan but more succinctly focuses on the pieces of information that are directly actionable. Figure 3 provides an example of the table summarizing patient risk profiles in *BNAV,* and Fig. 4 provides an example of the Provider Action Plan.

In order to fully prepare the provider for an informed discussion during the patient’s clinic visit, *BNAV* also provides educational modules on topics such as genetic testing, patient-centered care, and prevention options for *BRCA1/2* mutation carriers. These educational modules link to the evidence base that supports the covered topics in order to increase provider knowledge about genetic testing and facilitate shared decision making about a genetic counseling referral.

**Fig. 5:**
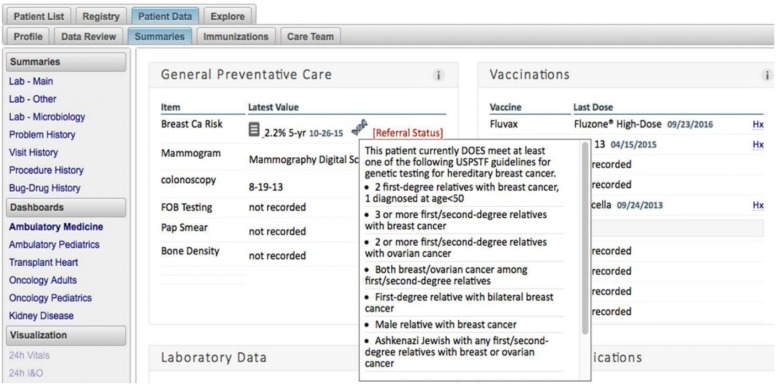
Example of the electronic health record (EHR)-embedded dashboard notice

Finally, the personalized risk information calculated by *RealRisks* is further integrated into clinical workflow through an alert that appears in the EHR-embedded dashboard used by the institution. This alert flags the patient as eligible for genetic testing and provides her five-year breast cancer risk. Figure 5 provides an example of this EHR-embedded dashboard alert.

### Control group

All participating patients will receive educational materials that include a brochure from our institution’s breast cancer prevention clinic and standard educational materials from the Susan G. Komen foundation on genes and breast cancer. Additionally, all patients will receive a letter that informs them of their eligibility for *BRCA1/2* genetic testing, outlines breast and ovarian cancer prevention options, and encourages them to discuss referral to genetic counseling with their health care provider. For providers, all enrolled patients will be flagged by the EHR-embedded dashboard alert.

### Study procedure

After providing informed consent, all patients will complete a baseline survey and then be sent the standard educational materials and eligibility letter. If the patients are in the intervention group, they will also receive a letter with instructions for accessing the *RealRisks* decision aid. Upon completing *RealRisks*, intervention patients will be encouraged to print their Patient Action Plan and bring it to their upcoming clinical appointments. All patients will complete another survey within 2 weeks of the date they are sent the educational materials or complete *RealRisks*, depending on the randomization arm to which they are assigned.

We will make appointments with our institution’s community resource center for patients who lack computer access. During these appointments, a member of the study team will assist the patient in accessing *RealRisks* and printing her action plan. Similarly, the study team will conduct phone interviews for participants who are not comfortable or able to self-administer the online questionnaires.

One week prior to an enrolled patient’s clinic visit, providers randomized into the intervention arm will be sent this patient’s Provider Action Plan and encouraged to visit the *BNAV* toolbox.

All patients and providers will complete additional questionnaires after their clinical encounter and at 6 months after baseline.

### Outcome measurement

The study’s primary endpoint is the appropriate uptake of HBOC genetic counseling, as assessed by electronic health record review at 6 months. Secondarily, we will assess the decision support tools’ effect on measures of decision quality, such as informed choice, decision conflict, and shared decision making. We will also explore which decision antecedents, such as genetic testing knowledge, breast cancer risk perceptions and worry, and decision self-efficacy, have an effect on these outcomes.

Table 1 provides a table outlining the measurement scales used at each evaluation. The primary and secondary outcomes of interest are also described below.

**Table 1:** Measures and Schedule of Evaluation

Patient Evaluations	Baseline	Post *RealRisks* or Educational Material	Post-Visit	6 M
Demographics	X			
Literacy [56]	X			
eHealth Literacy [57]	X			
Subjective Numeracy [58]	X			
Acculturation [59]	X			
Interest in Genetic Testing [71]	X			
Preparation in Decision Making	X	X		
Perceived Breast Cancer Risk [60]	X	X	X	X
Breast Cancer Worry [72]	X	X	X	X
Genetic Testing Knowledge [51]	X	X	X	X
Genetic Testing Attitudes [50]	X	X	X	X
Decision Self-Efficacy [73]	X	X		X
Decision Conflict [52]	X	X	X	X
Genetic Testing Decision		X	X	X
*RealRisks*/Education Material Comments		X		
Shared Decision Making [54]			X	
Trust in Health Care Providers [61]			X	
Decisional Regret [74]			X	X
Self-Reported Uptake of Genetic Counseling				X
Provider Evaluations	Baseline	Post-Visit	6 M	
Demographics	X			
Subjective Risk Communication Confidence [62]	X			
Confidence in Managing Patients w/ a Family History of Breast/Ovarian Cancer [63]	X		X	
Genetic Testing Knowledge [64]	X		X	
Genetic Testing Attitudes [75]	X		X	
Subjective Norms [65]	X		X	
Perceived Behavioral Control [65]	X		X	
Behavioral Intention [65]	X		X	
Orientation Towards Shared Decision Making [66]	X		X	
Shared Decision Making [55]		X		

#### Primary outcome

##### Genetic counseling uptake at six months

Six months after a patient is sent the educational materials with or without *RealRisks*, we will determine whether or not she has undergone genetic counseling by examining her medical record.

In order to avoid long wait times for genetic counseling appointments serving as a barrier to genetic counseling uptake, we have coordinated with our institution’s genetics clinic to ensure that study participants are seen within a few weeks of referral. We ask patients directly if they have undergone genetic counseling in the six-month questionnaire. We also ask providers if they choose to refer the patient to genetic counseling in their post-visit questionnaire.

#### Secondary outcomes

##### Informed choice

We will use the Multidimensional Measure of Informed Choice to determine whether a patient has made an informed decision to pursue *BRCA1/2* genetic testing [50]. An informed choice is classified as one that is made with adequate knowledge and that is consistent with the respondent’s attitudes. In this way, two groups are classified as having made an informed choice: 1) those who have adequate genetic testing knowledge score (> 50% correct) using a validated measure consisting of 11 true/false questions, [51] have a positive attitude towards testing according to our Likert scale attitude measure, which was adapted from the attitude measure used by Marteau et al., [50] and who decide to undergo testing 2) those with adequate genetic testing knowledge, a negative attitude towards genetic testing, and who decide not to undergo testing. We will use the knowledge and attitude scores collected after the clinical encounter and at 6 months to calculate informed choice, and we will assess differences between the intervention and control groups.

##### Decision conflict

We will use the low-literacy version of the Decision Conflict Scale to measure the degree of personal uncertainty that a patient experiences regarding the decision to undergo genetic testing [52, 53]. We will measure decision conflict among patients at baseline, 1 month, after the clinical encounter, and 6 months. Changes in decision conflict will be compared between the intervention and control groups.

##### Shared decision making

We will assess the extent to which the decision to undergo genetic testing actively involves both the patient’s and provider’s preferences and values by assessing shared decision making among patients, using the SDM-Q9, [54] and providers, using the SDM-Q-DOC, [55] after the clinical encounter. We will compare differences in shared decision making between the intervention and control groups.

#### Exploratory mediating/moderating factors

We will also explore whether an array of decision antecedents have any mediating or moderating effects on the primary or secondary outcomes. The patient characteristics that we will explore include the following: demographics, health literacy, [56, 57] numeracy, [58] acculturation, [59] perceived breast cancer risk and accuracy of risk perceptions, [60] breast cancer worry, genetic testing knowledge, [51] attitudes, [50] self-efficacy in decision making, [52] preparation in decision making, and trust in health care providers [61].

The provider measures that we will evaluate include the following: demographic and professional/practice characteristics, subjective risk communication confidence, [62] confidence in managing patients with a family history of breast or ovarian cancer, [63] genetic testing knowledge, [64] attitudes, [65] subjective norms, [65] perceived behavioral control, [65] behavioral intention, [65] and orientation towards shared decision making [66].

### Sample size calculation and statistical methods

Our goal is to recruit 76 providers and a total of 190 women. With a total sample size of 190 women (95 per arm), assuming a two-sided Type 1 error of 5%, a 20% drop-out rate (effective sample size of 76 per arm), and an intracluster correlation of less than 0.2, we will have greater than 90% power to detect a difference of a 5% rate of appropriate genetic counseling in the control arm (based upon our pilot data) and 30% in the *RealRisks/BNAV* intervention arm. We will have 80% power to detect an effect size of .50 difference on decisional conflict scores between the intervention and control arms, assuming a two-sided *p*-value set at 0.05 and an intracluster correlation of less than 0.1. Based on previous studies, this effect size (mean difference/standard deviation) is judged clinically important because effect sizes observed between those who make and those who delay decisions have ranged between 0.43 and 0.82 [67]. Agreement between patients’ and providers’ SDM-Q-9 and SDM-Q-Doc scores will be measured using the intraclass correlation coefficient (ICC) procedure, based upon the analysis of variance and the estimation of variance components. The higher the magnitude of this coefficient (range, − 1 to 1), the better the absolute match between the patient’s and provider’s scores.

Comparisons between the intervention and control groups will be conducted using Chi-square tests for categorical variables and Student’s t-tests for continuous variables. After generating descriptive statistics, we will conduct bivariate analyses using Chi-Square tests, t-tests, and Pearson correlation coefficients to determine associations between study variables and genetic counseling uptake rate. Depending on the scale of each outcome variable (continuous or binary) and the scale of the independent variable (categorical or continuous), we will use ANOVAs, linear regression, logistic regression, variance components analysis, mixed models, and generalized estimating equations (GEE) to identify variables that are associated with each outcome.

#### Trial status

We have begun recruiting and enrolling both patients and providers into the trial. After 5 months of recruitment and engagement, we have enrolled over 50 health care providers and 20 patients.

This trial is registered retrospectively in clinicaltrials.gov under trial number NCT03470402: 20 March 2018.

## Discussion

*BRCA1/2* mutations are the strongest known breast cancer risk factors, conferring up to a 60–80% lifetime risk of breast cancer among female carriers [4]. While this risk is significant, mutations in the *BRCA1* and *BRCA2* genes are actionable once identified. Cancer prevention options can reduce breast cancer risk by up to 90% [8] and all-cause mortality by up to 77% [16, 18]. Identifying those who are at increased risk of carrying a mutation and facilitating their access to genetic counseling and testing has the potential to significantly alleviate the public health burden of hereditary breast and ovarian cancers. For this reason, the USPSTF has recommended that primary care providers screen asymptomatic women for a family history indicative of a *BRCA1/2* mutation [21].

Nevertheless, while incorporating efficient family history screening into clinic workflow is critical to identifying patients eligible for *BRCA1/2* testing, collecting detailed family history is time consuming and difficult. We have found that providers at our institution often report insufficient time to complete a comprehensive family history intake or risk assessment during the clinical encounter, particularly when the patient presents with ongoing comorbidities that need to be managed [40]. While recruiting primary care providers into this trial, many were enthusiastic because they understood the importance of collecting and analyzing hereditary cancer information yet lacked the time to conduct such an assessment themselves. This lack of data collection is likely the principal reason behind our previous work’s finding that family history data is insufficiently recorded in our institution’s electronic health records [26].

*RealRisks* and *BNAV* have the potential to addresses this issue by aiding a patient in collecting her family history data, clarifying her values, and deliberating her choice to seek genetic testing *before* she sees her provider during her clinical visit. By doing so, *RealRisks* and *BNAV* can help to ensure that both the patient and her provider are better prepared to make a shared and informed decision about *BRCA1/2* genetic testing while reducing the time burden this conversation would normally bestow upon the rest of the clinical encounter.

This intervention may also have the potential to alleviate some of the disparities posed by the proliferation of precision prevention and genomic medicine. Although the prevalence of *BRCA1/2* mutations is comparable among most racial/ethnic groups except Ashkenazi Jews, racial/ethnic minorities and women from lower education or income levels are less likely to be referred to and receive genetic testing for these mutations [27, 28, 34, 68]. As advances in technology and genomic medicine lead to new innovations in cancer prevention, it is important to ensure that these innovations are accessible to all high-risk individuals in order to reduce health disparities. This study can help make progress in achieving this goal by evaluating tools that were designed to incorporate hereditary breast cancer risk assessment into routine primary care and to promote knowledge, accuracy of risk perceptions, and informed decision making in a diverse community with a high proportion of under-served, low-numerate, and high-risk patients.

The American Society for Clinical Oncology recommends that all oncology providers use family history information to inform clinical decision making [69]. While this recommendation underscores the importance of family history data in patient care, the data’s utility for prevention will remain limited if such risk assessments are only adopted by oncology specialists. *RealRisks* and *BNAV* can help to promote such practices in primary care by communicating risk information in a way that is integrated into clinic workflow. Furthermore, this study’s focus on patient-provider dyads in a diverse primary care setting—including various types of physicians and physician extenders practicing in internal medicine, family medicine, and gynecology—can help expand our understanding of how best to promote appropriate referrals for *BRCA1/2* genetic counseling from the primary care setting.

Additionally, as genetic testing services are becoming increasingly accessible and affordable, the patient preparation provided by *RealRisks* may allow for the more efficient use of genetic counseling and testing services by providing genetic counselors with a pre-collected and detailed pedigree.

Finally, while DAs are often shown to be efficacious in improving knowledge, risk perceptions, and decision confidence, they are insufficient in and of themselves in incorporating decision-making about genetic testing in the clinic [70]. In order to increase our tools’ adoptability and effectiveness, we have worked to integrate them into clinical workflow and existing clinical systems. The printable Patient Action Plan provides the patient with a tangible product that she can use to assert her decision making into the clinical encounter. The Provider Action Plan is sent to the provider via secure health message (SHM) before the patient’s clinical encounter. This prompts and prepares the provider for discussing genetic counseling with the patient, and the SHM ensures that the action plan’s patient-derived data is accessible through the institution’s EHR system. The EHR-embedded dashboard alert serves a similar purpose in prompting the provider and incorporating risk information into existing clinical tools. We are currently working on further integrating *RealRisks* with existing EHRs by developing a Fast Healthcare Interoperability Resources-standard application programing interface (FHIR API) to prepopulate risk information in *RealRisks* and return patient-derived data to the medical record.

Our future and related work focuses on ensuring access to proper downstream, risk management services, including psychosocial support, for patients with positive genetic test results.

In conclusion, we have developed informatics-based tools which collect, analyze, and communicate patient risk information in order to provide tailored educational resources to patients and their providers. These tools have the potential to mitigate common patient and provider barriers to breast cancer risk assessment and prevention. We are currently evaluating these tools’ effectiveness among patient and provider clusters in various primary care practices that serve a large and diverse patient population. We hope the results from this trial will help to inform the future implementation of personalized breast cancer prevention efforts.

## Abbreviations

ANOVA: Analysis of Variance
API: Application Programming Interface
BNAV: Breast Cancer Risk Navigation Toolbox
*BRCA1/2*: *BRCA1* and *BRCA2*
BSO: Bilateral Salpingo-Oophorectomy
EHR: Electronic Health Record
FHIR: Fast Healthcare Interoperability Resources
GEE: Generalized Estimating Equation
HBOC: Hereditary Breast and Ovarian Cancer
ICC: Intracluster Correlation Coeficient
MRI: Magnetic Resonance Imaging
PCP: Primary Care Provider
RCT: Randomized Controlled Trial
SHM: Secure Health Message
USPSTF: United States Preventative Services Task Force

## References

[1] Singletary SE (2003). Rating the risk factors for breast cancer. Ann Surg.

[2] Siegel RL, Miller KD, Jemal A (2016). Cancer statistics, 2016. CA Cancer J Clin.

[3] Anglian B (2000). Prevalence and penetrance of mutations in BRCA1 and BRCA2 in a population based series of breast cancer cases. Br J Cancer.

[4] Kuchenbaecker KB, Hopper JL, Barnes DR, Phillips K-A, Mooij TM, Roos-Blom M-J, Jervis S, Van Leeuwen FE, Milne RL, Andrieu N (2017). Risks of breast, ovarian, and contralateral breast cancer for BRCA1 and BRCA2 mutation carriers. Jama.

[5] Risch HA, McLaughlin JR, Cole DE, Rosen B, Bradley L, Fan I, Tang J, Li S, Zhang S, Shaw PA (2006). Population BRCA1 and BRCA2 mutation frequencies and cancer penetrances: a kin–cohort study in Ontario, Canada. J Natl Cancer Inst.

[6] Pal T, Permuth-Wey J, Betts JA, Krischer JP, Fiorica J, Arango H, LaPolla J, Hoffman M, Martino MA, Wakeley K (2005). BRCA1 and BRCA2 mutations account for a large proportion of ovarian carcinoma cases. Cancer.

[7] Moyer VA (2014). Risk assessment, genetic counseling, and genetic testing for BRCA-related cancer in women: US preventive services task force recommendation statement. Ann Intern Med.

[8] Rebbeck TR, Friebel T, Lynch HT, Neuhausen SL, Lvt V, Garber JE, Evans GR, Narod SA, Isaacs C, Matloff E (2004). bilateral prophylactic mastectomy reduces breast Cancer risk in BRCA1 and BRCA2 mutation carriers: the PROSE study group. J Clin Oncol.

[9] Saslow D, Boetes C, Burke W, Harms S, Leach MO, Lehman CD, Morris E, Pisano E, Schnall M, Sener S (2007). American Cancer Society guidelines for breast screening with MRI as an adjunct to mammography. CA Cancer J Clin.

[10] Warner E, Hill K, Causer P, Plewes D, Jong R, Yaffe M, Foulkes WD, Ghadirian P, Lynch H, Couch F (2011). Prospective study of breast cancer incidence in women with a BRCA1 or BRCA2 mutation under surveillance with and without magnetic resonance imaging. J Clin Oncol.

[11] Warner E, Messersmith H, Causer P, Eisen A, Shumak R, Plewes D (2008). Systematic review: using magnetic resonance imaging to screen women at high risk for breast cancer. Ann Intern Med.

[12] Isern A, Loman N, Malina J, Olsson H, Ringberg A (2008). Histopathological findings and follow-up after prophylactic mastectomy and immediate breast reconstruction in 100 women from families with hereditary breast cancer. Eur J Surg Oncol.

[13] Kauff ND, Domchek SM, Friebel TM, Robson ME, Lee J, Garber JE, Isaacs C, Evans DG, Lynch H, Eeles RA (2008). Risk-reducing salpingo-oophorectomy for the prevention of BRCA1-and BRCA2-associated breast and gynecologic cancer: a multicenter, prospective study. J Clin Oncol.

[14] Rebbeck TR, Kauff ND, Domchek SM (2009). Meta-analysis of risk reduction estimates associated with risk-reducing salpingo-oophorectomy in BRCA1 or BRCA2 mutation carriers. J Natl Cancer Inst.

[15] Domchek SM, Friebel TM, Neuhausen SL, Wagner T, Evans G, Isaacs C, Garber JE, Daly MB, Eeles R, Matloff E (2006). Mortality after bilateral salpingo-oophorectomy in BRCA1 and BRCA2 mutation carriers: a prospective cohort study. The lancet oncology.

[16] Domchek SM, Friebel TM, Singer CF, Evans DG, Lynch HT, Isaacs C, Garber JE, Neuhausen SL, Matloff E, Eeles R (2010). Association of risk-reducing surgery in BRCA1 or BRCA2 mutation carriers with cancer risk and mortality. Jama.

[17] Kaas R, Verhoef S, Wesseling J, Rookus MA, Oldenburg HS, Peeters M-JV, Rutgers EJ (2010). Prophylactic mastectomy in BRCA1 and BRCA2 mutation carriers: very low risk for subsequent breast cancer. Ann Surg.

[18] Finch AP, Lubinski J, Møller P, Singer CF, Karlan B, Senter L, Rosen B, Maehle L, Ghadirian P, Cybulski C (2014). Impact of oophorectomy on cancer incidence and mortality in women with a BRCA1 or BRCA2 mutation. J Clin Oncol.

[19] King M-C, Wieand S, Hale K, Lee M, Walsh T, Owens K, Tait J, Ford L, Dunn BK, Costantino J (2001). Tamoxifen and breast cancer incidence among women with inherited mutations in BRCA1 and BRCA2: National Surgical Adjuvant Breast and bowel project (NSABP-P1) breast Cancer prevention trial. Jama.

[20] Narod SA, Brunet J-S, Ghadirian P, Robson M, Heimdal K, Neuhausen SL, Stoppa-Lyonnet D, Lerman C, Pasini B, De Los RP (2000). Tamoxifen and risk of contralateral breast cancer in BRCA1 and BRCA2 mutation carriers: a case-control study. Lancet.

[21] Moyer VA, On behalf of the USPSTF (2014). risk assessment, genetic counseling, and genetic testing for brca-related cancer in women: U.S. preventive services task force recommendation statement. Ann Intern Med.

[22] Bellcross CA, Leadbetter S, Alford SH, Peipins LA. Prevalence and Healthcare Actions of Women in a Large Health System with a Family History Meeting the 2005 USPSTF recommendation for BRCA genetic counseling referral. Cancer Epidemiol Biomarkers Prev. 22(4):728–35. 10.1158/1055-9965.EPI-12-1280 PMC4759639 23371291

[23] Willis AM, Smith SK, Meiser B, Ballinger ML, Thomas DM, Young MA (2017). Sociodemographic, psychosocial and clinical factors associated with uptake of genetic counselling for hereditary cancer: a systematic review. Clin Genet.

[24] Ropka ME, Wenzel J, Phillips EK, Siadaty M, Philbrick JT (2006). Uptake rates for breast cancer genetic testing: a systematic review. Cancer Epidemiol Biomarkers Prev.

[25] Levy DE, Garber JE, Shields AE (2009). Guidelines for genetic risk assessment of hereditary breast and ovarian Cancer: early disagreements and low utilization. J Gen Intern Med.

[26] McGuinness JE, Trivedi MS, Vanegas A, Colbeth H, Sandoval R, Kukafka R, Crew KD: Decision support for family history intake to determine eligibility for BRCA testing among multiethnic women. In*.*: American society of Clin Oncol; 2017.

[27] Joseph G, Kaplan C, Luce J, Lee R, Stewart S, Guerra C, Pasick R (2012). Efficient identification and referral of low-income women at high risk for hereditary breast Cancer: a practice-based approach. Public Health Genomics.

[28] Shields AE, Burke W, Levy DE (2008). Differential use of available genetic tests among primary care physicians in the United States: results of a national survey. Genetics in medicine.

[29] Hall MJ, Olopade OI (2006). Disparities in genetic testing: thinking outside the BRCA box. J Clin Oncol.

[30] Yi H, Xiao T, Thomas PS, Aguirre AN, Smalletz C, Dimond J, Finkelstein J, Infante K, Trivedi M, David R: Barriers and facilitators to patient-provider communication when discussing breast cancer risk to aid in the development of decision support tools. In: *AMIA Annual Symposium Proceedings: 2015*: American Medical Informatics Association; 2015: 1352. PMC4765687 26958276

[31] Pal T, Cragun D, Lewis C, Doty A, Rodriguez M, Radford C, Thompson Z, Kim J, Vadaparampil ST (2013). A statewide survey of practitioners to assess knowledge and clinical practices regarding hereditary breast and ovarian cancer. Genet Test Mol Biomarkers..

[32] Culver JO, Bowen DJ, Reynolds SE, Pinsky LE, Press N, Burke W (2009). Breast cancer risk communication: assessment of primary care physicians by standardized patients. Genetics in Medicine.

[33] Trivers KF, Baldwin L-M, Miller JW, Matthews B, Andrilla CHA, Lishner DM, Goff BA (2011). Reported referral for genetic counseling or BRCA 1/2 testing among United States physicians. Cancer.

[34] Bellcross CA, Kolor K, Goddard KA, Coates RJ, Reyes M, Khoury MJ (2011). Awareness and utilization of BRCA1/2 testing among US primary care physicians. Am J Prev Med.

[35] Nair N, Bellcross C, Haddad L, Martin M, Matthews R, Gabram-Mendola S, Crane B, Meaney-Delman D (2017). Georgia primary care providers’ knowledge of hereditary breast and ovarian Cancer syndrome. J Cancer Educ.

[36] Suther S, Goodson P (2003). Barriers to the provision of genetic services by primary care physicians: a systematic review of the literature. Genetics in Medicine.

[37] Murff HJ, Greevy RA, Syngal S (2007). The comprehensiveness of family cancer history assessments in primary care. Public Health Genomics.

[38] Peters E, Hibbard J, Slovic P, Dieckmann N (2007). Numeracy skill and the communication, comprehension, and use of risk-benefit information. Health Aff.

[39] Guerra CE, Jacobs SE, Holmes JH, Shea JA (2007). Are physicians discussing prostate Cancer screening with their patients and why or why not? A pilot study. J Gen Intern Med.

[40] Yi H, Xiao T, Thomas PS, Aguirre AN, Smalletz C, Dimond J, Finkelstein J, Infante K, Trivedi M, David R (2015). Barriers and facilitators to patient-provider communication when discussing breast Cancer risk to aid in the development of decision support tools. AMIA Ann Symp Proc.

[41] Finkelstein J, Wood J, Crew KD, Kukafka R (2017). Introducing a comprehensive informatics framework to promote breast Cancer risk assessment and chemoprevention in the primary care setting. AMIA Summits on Translational Science Proceedings.

[42] Coe AM, Ueng W, Vargas JM, David R, Vanegas A, Infante K, Trivedi M, Yi H, Dimond J, Crew KD: Usability Testing of a Web-Based Decision Aid for Breast Cancer Risk Assessment Among Multi-Ethnic Women. In: *AMIA Annual Symposium Proceedings: 2016*: American Medical Informatics Association; 2016: 411. PMC5333260 28269836

[43] Kukafka R, Yi H, Xiao T, Thomas P, Aguirre A, Smalletz C, David R, Crew K (2015). Why breast Cancer risk by the numbers is not enough: evaluation of a decision aid in multi-ethnic, low-numerate women. J Med Internet Res.

[44] Alge D, on Behalf on the New York Presbyterian Ambulatory Care Network: Ambulatory Care Network: 2016 Annual Report. In*.*: New York Presbyterian Hospital 2017.

[45] Ancker JS, Weber EU, Kukafka R (2011). Effect of arrangement of stick figures on estimates of proportion in risk graphics. Med Decis Mak.

[46] Deci EL, Ryan R: RM (1985**):** Intrinsic motivation and self-determination in human behavior. N Y 1997.

[47] Elwyn G, Frosch D, Thomson R, Joseph-Williams N, Lloyd A, Kinnersley P, Cording E, Tomson D, Dodd C, Rollnick S (2012). Shared decision making: a model for clinical practice. J Gen Intern Med.

[48] Mazzola E, Blackford A, Parmigiani G, Biswas S: Recent enhancements to the genetic risk prediction model BRCAPRO. *Cancer informatics* 2015, 14:CIN. S17292. 10.4137/CIN.S17292 PMC4428390 25983549

[49] Vogel KJ, Atchley DP, Erlichman J, Broglio KR, Ready KJ, Valero V, Amos CI, Hortobagyi GN, Lu KH, Arun B (2007). BRCA1 and BRCA2 genetic testing in Hispanic patients: mutation prevalence and evaluation of the BRCAPRO risk assessment model. J Clin Oncol.

[50] Marteau TM, Dormandy E, Michie S (2001). A measure of informed choice. Health Expectations : An International Journal of Public Participation in Health Care and Health Policy.

[51] Lerman C, Narod S, Schulman K (1996). Brca1 testing in families with hereditary breast-ovarian cancer: a prospective study of patient decision making and outcomes. JAMA.

[52] O'Connor AM (1995). Validation of a decisional conflict scale. Med Decis Mak.

[53] Linder SK, Swank PR, Vernon SW, Mullen PD, Morgan RO, Volk RJ (2011). Validity of a low literacy version of the decisional conflict scale. Patient Educ Couns.

[54] Kriston L, Scholl I, Hölzel L, Simon D, Loh A, Härter M (2010). The 9-item shared decision making questionnaire (SDM-Q-9). Development and psychometric properties in a primary care sample. Patient Educ Couns.

[55] Scholl I, Kriston L, Dirmaier J, Buchholz A, Härter M (2012). Development and psychometric properties of the shared decision making questionnaire–physician version (SDM-Q-doc). Patient Educ Couns.

[56] Chew LD, Griffin JM, Partin MR, Noorbaloochi S, Grill JP, Snyder A, Bradley KA, Nugent SM, Baines AD, VanRyn M (2008). Validation of screening questions for limited health literacy in a large VA outpatient population. J Gen Intern Med.

[57] Norman CD, Skinner HA: eHEALS: the eHealth literacy scale. J Med Internet Res 2006, 8(4). 10.2196/jmir.8.4.e27 PMC1794004 17213046

[58] Zikmund-Fisher BJ, Smith DM, Ubel PA, Fagerlin A (2007). Validation of the subjective numeracy scale: effects of low numeracy on comprehension of risk communications and utility elicitations. Med Decis Mak.

[59] Marin G, Sabogal F, Marin BV, Otero-Sabogal R, Perez-Stable EJ (1987). Development of a short acculturation scale for Hispanics. Hisp J Behav Sci.

[60] Levy AG, Shea J, Williams SV, Quistberg A, Armstrong K (2006). Measuring perceptions of breast cancer risk. Cancer Epidemiology and Prevention Biomarkers.

[61] Hall MA, Zheng B, Dugan E, Camacho F, Kidd KE, Mishra A, Balkrishnan R (2002). Measuring patients’ trust in their primary care providers. Med Care Res Rev.

[62] Han PKJ, Joekes K, Elwyn G, Mazor KM, Thomson R, Sedgwick P, Ibison J, Wong JB (2014). Development and evaluation of a risk communication curriculum for medical students. Patient Educ Couns.

[63] Watson E, Clements A, Yudkin P, Rose P, Bukach C, Mackay J, Lucassen A, Austoker J (2001). Evaluation of the impact of two educational interventions on GP management of familial breast/ovarian cancer cases: a cluster randomised controlled trial. Br J Gen Pract.

[64] Marzuillo C, De Vito C, Boccia S, D’Addario M, D’Andrea E, Santini P, Boccia A, Villari P (2013). Knowledge, attitudes and behavior of physicians regarding predictive genetic tests for breast and colorectal cancer. Prev Med.

[65] Wilson BJ, Islam R, Francis JJ, Grimshaw JM, Permaul JA, Allanson JE, Blaine S, Graham ID, Meschino WS, Ramsay CR (2016). Supporting genetics in primary care: investigating how theory can inform professional education. Eur J Hum Genet.

[66] Krupat E, Rosenkranz SL, Yeager CM, Barnard K, Putnam SM, Inui TS (2000). The practice orientations of physicians and patients: the effect of doctor–patient congruence on satisfaction. Patient Educ Couns.

[67] O’Connor AM, Stacey D, Entwistle V, Llewellyn-Thomas H, Rovner D, Holmes-Rovner M, Tait V, Tetroe J, Fiset V, Barry M. Decision aids for people facing health treatment or screening decisions. Cochrane Database Syst Rev. 2003;2 10.1002/14651858.CD001431 12804407

[68] Armstrong K, Micco E, Carney A, Stopfer J, Putt M (2005). Racial differences in the use of brca1/2 testing among women with a family history of breast or ovarian cancer. JAMA.

[69] Lu KH, Wood ME, Daniels M, Burke C, Ford J, Kauff ND, Kohlmann W, Lindor NM, Mulvey TM, Robinson L (2014). American Society of Clinical Oncology expert statement: collection and use of a Cancer family history for oncology providers. J Clin Oncol.

[70] Elwyn G, Scholl I, Tietbohl C, Mann M, Edwards AGK, Clay C, Légaré F, Weijden Tvd, Lewis CL, Wexler RM *et al*: “many miles to go …”: a systematic review of the implementation of patient decision support interventions into routine clinical practice. BMC Medical Informatics and Decision Making 2013, 13(Suppl 2):S14-S14. 10.1186/1472-6947-13-S2-S14 PMC4044318 24625083

[71] Bowen DJ, Robbins R, Bush N, Meischke H, Ludwig A, Wooldridge J (2017). Effects of a web-based intervention on women’s breast health behaviors. Translational Behavioral Medicine.

[72] Lerman C, Kash K, Stefanek M (1994). Younger women at increased risk for breast cancer: perceived risk, psychological well-being, and surveillance behavior. J Natl Cancer Inst Monogr.

[73] O’Connor A: User manual-decision self-efficacy scale. *Patient Decision Aids, Ottawa Hospital Research Institute (OHIR) Web site https://decisionaid ohri ca/docs/develop/Tools/Decision_SelfEfficacy pdf (accessed 4 Feb 2015)* 1995.

[74] Brehaut JC, O'Connor AM, Wood TJ, Hack TF, Siminoff L, Gordon E, Feldman-Stewart D (2003). Validation of a decision regret scale. Med Decis Mak.

[75] Bouhnik A-D, N’Diaye K, Evans DG, Harris H, Tibben A, van Asperen C, Schmidtke J, Nippert I, Mancini J, Julian-Reynier C (2017). Validation of a scale for assessing attitudes towards outcomes of genetic cancer testing among primary care providers and breast specialists. PLoS One.

